# The Tails of Two Avian Schistosomes: Paired Exposure Study Demonstrates *Trichobilharzia stagnicolae* Penetrates Human Skin More Readily than a Novel Avian Schistosome from *Planorbella*

**DOI:** 10.3390/pathogens11060651

**Published:** 2022-06-04

**Authors:** Nathaniel J. Anderson, Curtis L. Blankespoor, Randall J. DeJong

**Affiliations:** 1 Department of Biology, Calvin University, Grand Rapids, MI 49546, USA; nathan.anderson276@gmail.com; 2 Science Department, Jackson College, Jackson, MI 49201, USA; blankescurtis@jccmi.edu or; 3 University of Michigan Biological Station, Pellston, MI 49769, USA; 4 Swimmer’s Itch Solutions LLC, Adrian, MI 49221, USA

**Keywords:** *Planorbella*, Helisoma, avian schistosome, swimmer’s itch, cercariae behavior, skin penetration, *Trichobilharzia*, cercarial dermatitis

## Abstract

A novel schistosome from *Planorbella* snails currently known as avian schistosomatid sp. C (ASC) was recently described as being capable of causing the papules associated with swimmer’s itch. We conducted a paired study with 24 human volunteers, exposing each of their forearms to five drops of water containing cercariae of ASC or *Trichobilharzia stagnicolae,* and examined the skin for papules 1–3 days later. A mixed effects model showed that only the parasite species significantly affected the number of papules, while prior experimental exposure, swimming history, and swimmer’s itch experience did not. The total number of papules produced by the two species were very different: ASC produced a total of 2 papules from the 298 cercariae used, compared to 49 papules from 160 *T. stagnicolae* cercariae, a difference factor of more than 43X, which was comparable to the odds ratio of 45.5 computed using the statistical model. A well-known agent of swimmer’s itch, *T. stagnicolae,* is able to penetrate human skin more frequently than ASC, likely meaning that ASC is only a minor cause of swimmer’s itch where *T. stagnicolae* is present. We also completed limited experiments that compared the cercarial behavior of the two species *in vitro* and in situ. A known stimulant of schistosome cercarial penetration, α-linolenic acid, did not stimulate ASC cercariae to initiate penetration-associated behaviors as frequently as *T. stagnicolae*. However, when placed on esophageal tissue of the known vertebrate host for ASC, Canada goose (*Branta canadensis*), ASC cercariae were observed penetrating the esophageal epithelium quickly, whereas *T. stagnicolae* cercariae did not exhibit any penetration behaviors.

## 1. Introduction

Cercariae of avian schistosomes were first described in 1928 as the causative agents of an irritating condition known as cercarial dermatitis, or swimmer’s itch, on Douglas Lake, Michigan [1]. Nearly 100 avian schistosome species have been identified around the globe, infecting snails of many different families, including Planorbidae [2,3]. Many planorbid snail genera have been found to be hosts to avian schistosomes, but there were no verified accounts of avian schistosomes in the genus *Planorbella (Helisoma)* until recently [4,5]. McPhail et al. [5] described the cercariae of novel species avian schistosomatid C (ASC), and used molecular data to link it to its definitive host, Canada goose (*Branta canadensis*).

Due to the widespread range of its snail and avian hosts, and its co-presence in inland Michigan lakes with other avian schistosome species well-known to cause swimmer’s itch (*Trichobilharzia stagnicolae, T. physellae,* and others) [6,7,8,9], we aimed to better understand to what degree ASC contributes to swimmer’s itch. McPhail et al. [5] exposed four individuals to multiple cercariae of this parasite and reported that one person developed a single papule. In this study, we exposed human volunteers to cercariae of ASC and *T. stagnicolae,* the dominant avian schistosome in many Michigan inland lakes [6,7,9,10,11], to measure and compare rates of papule formation. In addition, we completed limited observational studies comparing cercarial behaviors of the two species.

## 2. Results

### 2.1. DNA Sequences 

DNA sequences of 18S, 28S, and COI from cercariae of several *Planorbella* snails (GenBank ON303302, ON303651-652, ON303478) all verified that the cercariae being used were schistosomes, and matched those deposited for ASC [4,5].

### 2.2. Exposure Experiment 

**Figure 1:**
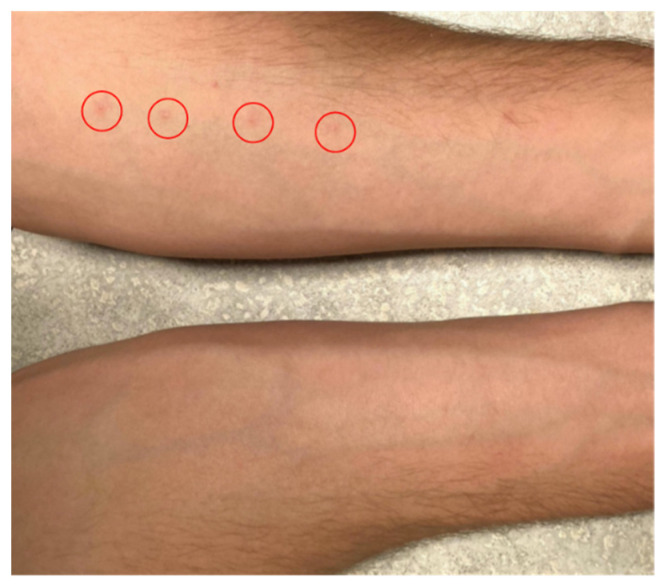
Arms of volunteer at post-exposure follow-up visit. Left arm (top), exposed to five *T. stagnicolae* cercariae, has four visible papules. Right arm (bottom), exposed to at least 10 ASC cercariae, did not develop any papules.

**Figure 2:**
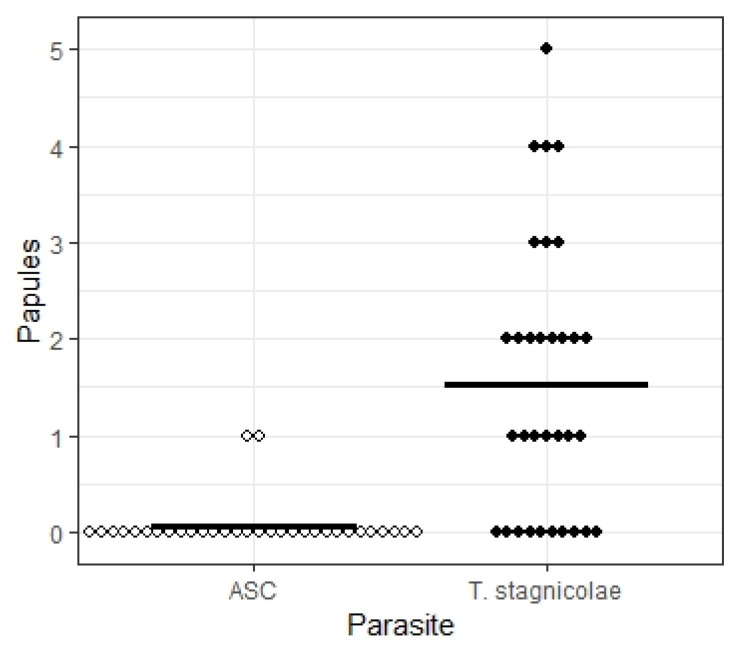
Number of papules produced on the arms of volunteers by five drops of cercariae-containing water. For *T. stagnicolae,* each drop contained 1 cercaria. For ASC, the first four participants received 1 cercaria per drop, but this was increased to 2–3 per drop for all other exposures to increase the chances of demonstrating that ASC can cause swimmer’s itch. Each rhombus represents the number of papules out of five that were observed on an individual arm (open = ASC, filled = *T. stagnicolae*). Horizontal line indicates mean number of papules per person (0.06 ASC, 1.53 *T. stagnicolae*).

Striking differences were observed between the two species in the number of papules they produced (Figure 1 and Figure 2). When exposed to five water drops that each contained a single *T. stagnicolae* cercaria, 15 of 24 (62.5%) study participants developed between 1–5 papules (median 2.4 papules ± 1.5 interquartile range). The same participants were exposed to five drops that each contained 1-3 ASC cercariae (see methods), but only one individual had a single ASC papule (this individual had two *T. stagnicolae* papules). Eight individuals who reacted to *T. stagnicolae* in the first exposure volunteered for a second exposure more than 10 days later, and the results were similar: seven (87.5%) developed 1–4 papules to *T. stagnicolae* (median 1.9 papules ± 1.0 interquartile range). Only one second exposure volunteer developed an ASC papule, along with two *T. stagnicolae* papules. This individual was not the same one who developed an ASC papule in the first exposure.

The data were first analyzed by a statistical model that treated each drop as an opportunity for papule formation, with a minimum of 0 and maximum of 5 for each individual. The effects of previous swimmer’s itch experience, inland lake swimming experience, first vs. second exposure, and parasite species were tested on the probability of papule formation using a mixed effects logistic regression, with the individual exposed as a random effect. Only parasite species was significant (X^2^ = 25.4828, *p* = 4.45 × 10^−7^). Odds ratio computation indicated that the odds of papule formation decreased by a factor of 45.5 for ASC compared to *T. stagnicolae* (95% CI: 10.3, 200.5).

**Figure 3:**
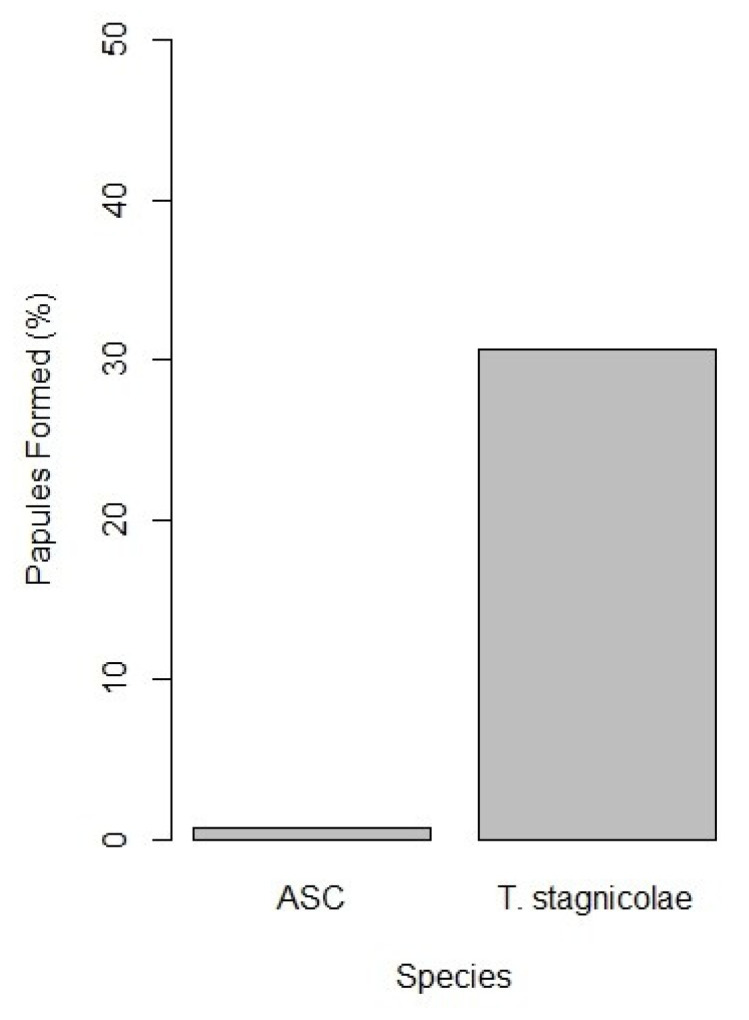
Percentage of cercariae of each species that induced papules in 32 exposures of human volunteers. *Trichobilharzia* *stagnicolae* induced 49 papules from 160 cercariae (30.6%). The recently described species, ASC, induced 2 papules from 298 cercariae (0.7%).

Each cercaria pipetted onto an arm (rather than each drop) can be viewed as an opportunity for penetration and papule development, and analyzing the data this way also shows a conspicuous contrast. Using the minimum number of ASC cercariae each volunteer was exposed to (5 for 4 of the exposures, 8 for 1 exposure, and 10 for 27 exposures, ASC cercariae produced a much lower (43X) number of papules (2 of 298, 0.7%) than did *T. stagnicolae* (49 of 160, 30.6%, Figure 3). Though it is possible that more than one ASC cercariae penetrated in the two ASC papules, the very low frequency of ASC penetration overall suggests that this is unlikely. 

While macules (reddened spots without protuberance) are sometimes described in the hours and even minutes after penetration [2,12,13,14], we observed only papules (reddened spots with protuberance), probably because our observations were not made until 1–3 days post-exposure. We were only able to measure the sizes of a small number of papules to give a representation of papule size: papule diameter ranged from 1.2 to 12.0 mm for *T. stagnicolae* (n = 10, mean 3.61 ± 2.77 mm) and the two ASC papules were 1.4 and 8.2 mm.

There was strong association between papule formation and the report of itching by study participants, suggesting that absence of ASC papules was likely due to lack of penetration, rather than lack of a host reaction. Of the 15 individuals developing *T. stagnicolae* papules from the first exposure, 14 reported itching on the *T. stagnicolae*-exposed arm only. Two of the nine individuals who did not develop papules reported brief mild itching, and only on the arm exposed to *T. stagnicolae*. The individual who developed papules from both species reported itching on both arms. Of those individuals who underwent a second exposure, five of seven who developed *T. stagnicolae* papules reported itching, again on the *T. stagnicolae*-exposed arm only. The individual who developed papules from both species in the second exposure reported itching for the arm exposed to *T. stagnicolae*, but not the arm exposed to ASC.

### 2.3. In Vitro Experiments 

Exposure results and differences in cercariae behavior, that were noticed while shedding snails, prompted us to further compare the penetration behaviors of the two parasite species, using established methods employing agar in microplate wells [15,16,17,18] and α-linolenic acid, a known penetration stimulant of avian and human schistosome cercariae [15,16,19]. Differences between the species were observed in penetration-related behaviors. A first experiment continuously monitored cercariae in wells, and body-tail separation was similar in the two species: 19 of 24 (79%) *T. stagnicolae* cercariae, and 15 of 17 (88%) ASC cercariae. However, tail separation was accompanied by strong differences in other behaviors, particularly enzyme secretion and motion. All 24 cercariae of *T. stagnicolae* secreted enzymes and became motionless within 20 min, whereas only 1 of 17 ASC cercariae showed enzyme secretion, and only 3 of 17 were motionless after 60 min. Likewise, a second experiment used slightly larger numbers of cercariae distributed in wells that were observed every 10 min: in these, all *T. stagnicolae* cercariae (n = 84) lost activity within 60 min, and enzyme secretion appeared to be ubiquitous, whereas only 16 of 36 (44%) ASC cercariae had lost activity within 60 min, and enzyme secretion was infrequent. These experiments were limited by the lifespan of the infected snails and the number of cercariae that could be obtained.

### 2.4. In Situ Experiment 

Due to the observed differences in penetration behavior and papule formation, we hypothesized that the ASC cercariae may enter the Canada goose host by being ingested during drinking or feeding, and then penetrating the soft tissue of the esophagus or other epithelia of the mouth. We were able to conduct a preliminary experiment using goose esophagus dissected from legally harvested Canadian geese. Though the experiment was limited, in situ cercariae behavior and penetration success on goose esophagus differed markedly between the two species. Briefly, we observed an immediate increase in ASC cercariae swimming movement, which quickly transitioned to creeping on the esophageal surface. Creeping duration varied, but once penetration was initiated, the cercarial body disappeared into the tissue within two minutes. Enzyme secretion by cercariae before penetration of the esophagus could not be seen under the optical conditions, but presumably occurred. Of the 16 ASC cercariae that we observed on the esophagus, 13 successfully penetrated goose esophageal epithelium in 10 min or less. The three ASC cercariae that did not penetrate appeared to be caught in a clump of debris and continued swimming or creeping without penetrating. In contrast, none of the 17 *T. stagnicolae* cercariae observed on goose esophagus attempted penetration or showed any behaviors that typically lead to penetration; their swimming movements appeared to be unaffected and creeping behavior was not initiated. This experiment was limited by the lifespan of the infected snails and the number of cercariae that could be obtained.

## 3. Discussion

The paired exposure design produced results that are consistent with ASC cercariae being less likely than *T. stagnicolae* to produce papules in humans. Of 32 total exposures, only 2 volunteers developed a papule from ASC cercariae (0.7% of at least 298 cercariae placed on arms), whereas 22 exposures resulted in 1–5 papules each from *T. stagnicolae* (49 total papules, 30.6% of 160 cercariae placed on arms), a difference factor of 43X, which was similar to the odds ratio of 45.5 computed by the statistical model. The low number of ASC papules in our study is consistent with the single papule reported in McPhail et al. [5].

The statistical model also showed that the effects of first vs. second exposure, prior inland lake swimming, and prior swimmer’s itch experience were not significant. These results are consistent with prior studies that show that even naive individuals usually have detectable papules [12,13,14], though previous exposure increases the size of the papules and the intensity of the host response [13,14]. Four individuals in our study with a known history of swimmer’s itch did not develop papules to either species, likely due to a lack of cercarial penetration. In addition, eight individuals in our study volunteered for a second exposure, with no measured increase in the number of developed papules for *T. stagnicolae.* The two instances of ASC papule development occurred in a re-exposed individual and in an individual with known swimmer’s itch history. However, in seven of eight re-exposed individuals, no ASC papules developed. 

While it is possible that our pipet transfer technique inadvertently prevented volunteers from being exposed equally to *T. stagnicolae* and ASC, this is an unlikely source of significant error. The steps we took to siliconize the Pasteur pipettes, check them for residual cercariae after each transfer, and monitor cercarial vitality following ejection from the pipettes all suggest that nearly all cercariae were successfully transferred. In addition, we have significant evidence to suggest that the cercariae retained vitality after transfer. The in situ experiment demonstrated that not only were the ASC cercariae fully intact and motile following transfer, but that they penetrated the esophageal epithelium of their definitive host. 

We attempted to mimic natural contact between parasite and swimmer by allowing drops containing cercariae to dry on the skin. However, it may not be possible to precisely mimic the conditions and stimuli which contribute to the stimulation of penetration and its success. For instance, the immersion of skin in water may, depending on the duration, soften the skin and/or change the composition and concentration of compounds on and in the skin. While the difference in papule formation between the species could partially be an artifact of the exposure method, we do not have a specific reason to suspect this. Such a large difference seems likely to persist in natural conditions, although it is equally plausible that it could become lesser (i.e., ASC penetration increases under natural conditions) or greater (i.e., *T. stagnicolae* penetration success may be even higher under natural conditions while ASC penetration success remains low). 

There are a limited number of studies that report the results of controlled exposure of multiple individuals to avian schistosomes. The percentage of volunteers in our study who showed visible reaction to *T. stagnicolae* (62.5%) was not quite as high as the rates in the literature, which could be due to the low number of cercariae used per person (five) limiting the number of penetration opportunities. Using 10–40 *T. stagnicolae* cercariae per person, Olivier [12] reports that ‘almost every’ one of 100 exposures to 30 individuals resulted in a visible reaction, while a second study [13] had 33 of 34 people react to first-time exposures of *T. stagnicolae* on their forearms. Cort [1] found five of seven individuals reacted to exposure to *T. physellae* with an undisclosed number of cercariae. When 10 workers were accidentally exposed to *T. szidati* cercariae in a small artificial water body, Macháček et al. [14] documented that all 10 developed multiple papules, including those without swimmer’s itch experience. We recommend that future studies which aim to determine the percentage of people that react use a higher number of cercariae, if permitted by their regulatory body for human subject research.

Even fewer studies investigate the capacity of more than one species to cause swimmer’s itch. To our knowledge, this study is the first to use a paired design to compare the penetration success on human skin by avian schistosome cercariae of different species simultaneously on the same individual. Haas and Haeberlein [15] compared cercarial behavior and penetration of human skin by the avian schistosome *T. szidati* with the human schistosome *Schistosoma mansoni,* but the individuals exposed were limited to the investigators themselves. Żbikowska [20] exposed small sets of volunteers sequentially to *Bilharziella polonica* and *T. szidati*, and concluded that *B. polonica* is an avian schistosome that does not cause swimmer’s itch. 

Though we did not initially set out to determine the cause of any difference between the two species, we provide preliminary evidence that the low frequency of ASC papule development may be due to differences in behavior and penetration ability. Observations of the cercariae in water revealed differences in behavior and longevity, and the in situ experiment showed that ASC was stimulated to, and did, quickly penetrate esophageal epithelium from its definitive host (Canada goose). It is interesting that *T. stagnicolae* was not stimulated to penetrate Canada goose esophagus at all, which might be explained by the incorrect host or that this species does not enter its known host, Common merganser (*Mergus merganser*), by the esophagus and instead penetrates the skin. Another species efficient at penetrating human skin, and a frequent cause of swimmer’s itch, *T. szidati,* exhibits cercarial behaviors that increase its likelihood of coming into contact with the webbed feet of waterfowl, and is stimulated by lipids from the foot skin [21,22]. Transmission to bird hosts via penetration of skin has been demonstrated in the lab for some species of avian schistosomes, including *T. szidati* [23,24,25]*,* as has infection through drinking water containing cercariae [23,26]. We emphasize that our results here are not only limited by the number of cercariae observed, but also that we have only shown ASC penetration of the dissected tissue, and not successful transmission to a live host.

In vivo experiments in this study suggest that α-linolenic acid, a representative of the free fatty acids known to stimulate penetration in *S. mansoni, S. japonicum, S. spindale,* and *T. szidati* [15,16,19,27], stimulates behaviors that lead to penetration by *T. stagnicolae*, but ASC cercariae are much less responsive. Again, variation among schistosome species is to be expected. Interestingly, Haas and van de Roemer [19] found that *T. szidati* was more stimulated by fatty acids from human skin than fatty acids of the natural waterfowl host. An important implication of our results is that the presence of certain avian schistosome species in a water body may not lead to frequent or widespread outbreaks of swimmer’s itch, whereas other species may be more prone to cause them. In addition, methods such as qPCR [6,28,29,30,31], that attempt to measure the number of cercariae in the water column, should not assume that all cercariae measured pose the same amount of risk to swimmers. Since each species infects a particular avian host, each is likely to have both general and host-specific adaptations for contact, stimulation, and success of penetration. These host-specific adaptations are likely to result in variation among species in their propensity and ability to penetrate the non-target of human skin. While the results of the current study confirm that ASC can cause papules in humans, the starkly lower rate of papule formation, and the observed cercarial behavior differences, suggest that it may do so less often than other avian schistosome species. 

The extensive geographic range of both the snail and waterfowl hosts for ASC, and its detection in Canada (Alberta) and the northern United States (Michigan, and now Wisconsin) by snail surveys in this study and others [4,5,31], raise the question of why schistosomes were not previously described from *Planorbella.* McPhail et al. [5] state that *Planorbella* have been less frequently included in snail surveys targeting schistosome discovery, and this may especially be the case where other avian schistosome species were already implicated in swimmer’s itch or identified as the dominant species [6,7]. It is also possible that since some schistosome species are discovered in association with a swimmer’s itch outbreak [3], the apparently diminished capacity of ASC to cause swimmer’s itch has helped it evade detection. Outside of already understood swimmer’s itch situations, *Planorbella* are among the most surveyed of all snails for trematodes, because of their geographic range, abundance, and frequent role as host for a high diversity of trematodes. The lack of prior schistosome discovery in *Planorbella* is especially remarkable given the number of snails and locations examined (e.g., California [32], Indiana [33], Michigan [34,35,36], New Jersey [37], North Carolina [38,39,40,41], Ontario [42], and Wisconsin [43]). We note that Cort reported schistosome cercariae from *Planorbella* in Michigan on two occasions [34,35], though it is possible that these were later determined to be spirorchids, as happened for a cercaria he described from *Stagnicola* [1,44]. Another non-conflicting possibility is that ASC is expanding its geographic range and/or becoming more common, coinciding with dramatic population increase and range expansion by the Canada goose [45]. 

Extensive geographic range for ASC and its abundance in the environment [5,31]) also suggests that swimmer exposure to ASC may be extensive [5,31]. The number of swimmer’s itch cases ASC produces will vary locally and will be strongly influenced by the abundance of *Planorbella*, the number of resident and migrating Canada geese, and its success in penetrating human skin. The results of the exposure study described here suggest that where ASC coexists with other schistosome species that are excellent penetrators of human skin and/or well-established as frequent causes of swimmer’s itch outbreaks, it is likely to play a secondary, minor role in producing swimmer’s itch cases.

## 4. Materials and Methods 

### 4.1. Snail Collection, Isolation, and Housing 

*Stagnicola emarginata* and *P. trivolvis* snails were collected by wading or snorkeling in June and July 2021 in Black Lake (Cheboygan County, MI, USA), Larks Lake (Emmet County, MI, USA), Walloon Lake (Leelanau County, MI, USA), and North and South Twin Lakes (Vilas County, WI, USA). All snails were housed in complete darkness overnight in buckets with fresh lake water. The following morning, the snails were individually isolated in small cups filled with well water and exposed to light for a minimum of 1 h, to stimulate the emergence of cercariae [46]. Furcocercous cercariae visually identified as avian schistosomes (pigmented ocelli, lack of finfolds [47]) were preserved in DNA-grade ethanol, and the host snails were transferred to aquaria for long-term maintenance. A small number (1–6) of ASC-infected *P. trivolvis* were found at each lake and *T. stagnicolae*-infected *S. emarginata* were found at Larks Lake, Walloon Lake, and North Twin Lake. Snails were housed in 20 L plastic bins filled with artificial pond water (APW) [48]. Under-gravel filters were covered by a 1.5 cm layer of gravel followed by a 3 cm layer of sand from collection sites. Snails were fed lettuce *ad libitum* by embedding the stalk in the sand, and detritus was siphoned from the bottom bi-weekly. 

### 4.2. DNA Extraction and Sequencing 

We extracted DNA from the preserved cercariae (Qiagen DNEasy blood/tissue kit), and used published primers to amplify regions of the 18S rRNA by PCR (primers 18JVSQF, 18SJVSQR), 28S rRNA (28SJVSQF, 28SJVSQR, C1, D2) [28,49], and COI genes (HCO, LCO) [50]. PCR products were purified with Exo-SAP-IT™ and submitted for Sanger sequencing. 

### 4.3. Volunteer Recruitment 

**Figure 4:**
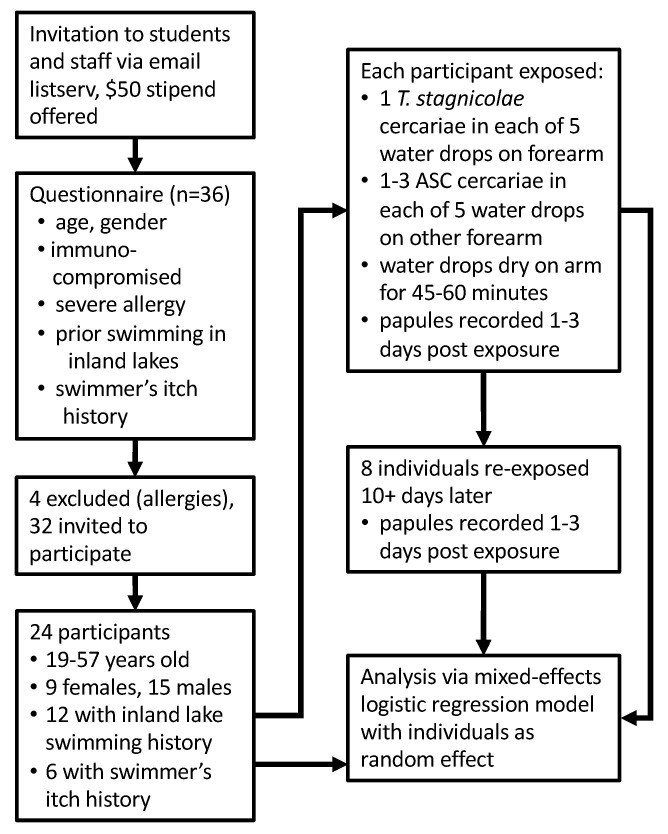
Study flow diagram. The recruitment, exclusion, and inclusion of volunteers, descriptive data of participants, and the methods of exposure. Data from the questionnaire (inland lake swimming history, swimmer’s itch history) and the results from exposures (number of papules formed from five drops) were used in the statistical model.

Procedures for recruitment are outlined in Figure 4. Volunteers from Calvin University were recruited for the study via an announcement on campus email listservs. Interested candidates were asked to complete a 10-item questionnaire requesting age, gender, health (especially any possible compromised immune status) and allergy information, prior swimmer’s itch experience, and inland lake swimming history (the Great Lakes have very little swimmer’s itch, so were not included). As a precaution, four candidates were excluded from the study because they declared a health or allergy condition that might have increased their risk of a severe reaction. Thirty-two candidates remained and were invited to participate, with 24 individuals (ages 19 to 57; 9 females and 15 males) responding and participating. Of those, 12 participants reported past swimming in Michigan inland lakes, and 6 participants reported a previous experience with known or probable swimmer’s itch. Study participants received a $50 stipend.

### 4.4. Exposure Procedures

Aquaria of infected snails were covered with dark plastic overnight to prevent premature light exposure. In the morning, the snails were isolated in untreated 6-well tissue culture plates and exposed to light for 1 h. Emergent cercariae were then used in human exposures at 1–6 (mean 2.9 ± 1.5) h, after the snails were first exposed to light. Preliminary observations of the ASC parasite indicated that they were prone to adhere to surfaces with their oral and ventral suckers, and were more likely to stick to Pasteur pipettes than *T. stagnicolae*. To reduce the rate at which both species stuck to pipettes, we used glass pipettes siliconized with Sigmacote®. In addition, we deposited small numbers of cercariae onto a watch glass under a dissection microscope, so that we could accurately determine the number of cercariae drawn into the pipette before transferring water drops onto participant skin. 

Study participants were asked to sit between two microscopes, with their forearms supine on the table in front of them. We used siliconized glass pipettes to transfer five drops of water containing cercariae onto each arm. Drops containing *T. stagnicolae* were placed on one arm, and ASC on the other arm. After each transfer, the pipette was checked under the microscope to ensure the cercariae had been transferred. For the first four volunteers exposed, each ASC drop contained one cercaria. To increase the number of opportunities for ASC cercariae to penetrate, each drop contained 2–3 ASC cercariae, which meant that volunteers were exposed to 10–15 ASC cercariae in all subsequent exposures. Drops containing *T. stagnicolae* contained a single cercaria each for every trial. One exposure was limited to four drops of ASC cercariae, due to insufficient viable cercariae. Study participants remained seated for 45–60 min, to allow the drops to evaporate completely.

Participants returned for follow-up observation 24–72 h later. The number of papules on each arm was recorded, and non-identifying photos were taken (e.g., Figure 1). All participants were asked if they experienced any itching, when they first noticed papules appearing, whether papules had increased or decreased in size over time, and if they had applied any anti-itch creams. To determine if prior exposure might increase the number of responses to ASC cercariae, participants who developed papules were also asked if they were willing to return for a second exposure. Eight participants agreed to be exposed a second time, 10–20 days after their first exposure, and to return for follow-up observation. Calipers were used to measure papule diameters for 10 random participants over the course of the study. We fitted a mixed-effects logistic regression model to predict the probability of papules as a function of first vs. second exposure, parasite species, inland lake swimming experience, and previous swimmer’s itch experience, with the random effect of the individual. Models were fitted with R version 4.0.4 using package glmmTMB [51,52].

### 4.5. In Vitro Behavior Experiments

We used established methods [15,16,17,18] to observe whether cercariae exhibited penetration behaviors in the presence of linolenic acid, a typical penetration stimulant of avian and human schistosomes [15,16,19]. Briefly, α-linolenic acid was incorporated at a concentration of 10 µM into agar (7.0 pH, 5 mM phosphate buffer), and layered into flat-bottom 96-well tissue culture plates. Preliminary trials indicated that clear penetration-associated behaviors, as defined by the above authors (creeping along the surface using suckers, enzyme secretion (excreted contents of penetration glands could typically be observed on the agar in these experiments), ‘pushing’ against the agar, tail separation, and eventual halting of all movement), occurred more frequently in *T. stagnicolae* than in the ASC. However, cercariae of both species were unable to physically penetrate the agar, despite varying agar concentrations from 2.5% [15] to 0.25%.

The following experiment was therefore conducted using conventional 1% agar with 10 µM α-linolenic acid, and by observing the above penetration-associated behaviors. Twenty-four *T. stagnicolae* and 17 ASC cercariae were pipetted into two separate wells, and behaviors were observed continuously for 60 min. A larger number of cercariae (n = 84 *T. stagnicolae* and n = 36 ASC cercariae) were pipetted into 4–5 additional wells each, and checked incrementally every 10 min for 60 min. All cercariae which exhibited penetration behaviors quickly lost all activity and became motionless, apparently having spent their enzymes and energy reserves during attempted penetration. In contrast, cercariae that did not exhibit these penetration behaviors retained swimming activity.

### 4.6. In Situ Behavior Experiment 

A local hunter donated two wild Canada geese, legally harvested as game, and we dissected the esophagus from each specimen. Each esophagus was cut into sections of approximately 5 cm in length, opened, cut in half longitudinally, and the debris rinsed away with APW. One half of each section was randomly assigned to a parasite species. After a water drop containing 1–5 cercariae was applied, the drop was observed under a dissection microscope. Cercarial eyespots allowed us to locate and track individual cercariae, though not all cercariae were relocated on the esophagus (some were carried to the side by water flow). The data reported only represents cercariae that were relocated on the esophagus and observed from the time of water drop application until penetration or 20 min, whichever came first. Cercariae were monitored for swimming and creeping behavior in addition to penetration of tissue. 

## 5. Conclusions

We tested the ability of the cercariae of two avian schistosome species to penetrate and cause swimmer’s itch papules in human skin, by exposing volunteers to both species simultaneously. The recently described species from the snail genus *Planorbella,* currently known as avian schistosomatid sp. C, caused significantly fewer papules than a well-known causal agent of swimmer’s itch, *T. stagnicolae*, likely due to differences in ability to penetrate human skin. It should not be surprising that avian schistosomes vary in their propensity to penetrate human skin and cause swimmer’s itch, given that each are adapted to different hosts and possibly different points of entry. 

## Data Availability

Summary data are available within the article. Anonymized dataset is available on request from the corresponding author, as allowed by the IRB.

## Institutional Animal Care and Use Committee Statement

The use of hunted Canada goose was reviewed and approved by the Institutional Animal Care and Use Committee (IACUC) of Calvin University, protocol BR2021-02.
